# Epidemiology of Chronic Suppurative Otitis Media: Systematic Review To Estimate Global Prevalence

**DOI:** 10.1007/s44197-025-00396-9

**Published:** 2025-04-03

**Authors:** Anjola Onifade, Henriette Wa Katolo, Siddharth Mookerjee, Mahmood F. Bhutta

**Affiliations:** 1 Department of ENT, University Hospitals Sussex, Brighton and Hove, UK; 2 Department of ENT, Hull University Hospitals, Hull, UK; 3 Dept Global Health and Infection, Brighton and Sussex Medical School, Brighton, UK

**Keywords:** Chronic suppurative otitis media, Global burden of disease, Health disparities, Low- and middle-income Countries, Hearing loss epidemiology, Public health interventions

## Abstract

**Background:**

Chronic Suppurative Otitis Media (CSOM) is a disorder characterised by a perforation of the tympanic membrane leading to ear discharge and hearing loss, a disability causing marginalisation in employment, education, social stigma, and reduced wellbeing and quality of life. Previous studies on the global epidemiology of this condition, despite methodological limitations, have estimated global prevalence at 200 million.

**Methods:**

Adhering to PRISMA guidelines, published literature was systematically reviewed across Ovid and Embase databases, with original community-based studies on CSOM published from 2004 to March 19th, 2025 extracted for final consideration on quality and relevance. Studies focusing on hospital populations were excluded as they seem more likely to represent a biased group of severe cases. Published articles were analysed for data on CSOM prevalence and associated risk factors.

**Results:**

From 5,394 articles, 29 cross-sectional studies met the inclusion criteria. Included studies predominantly originated from low- and middle-income countries (LMICs) and focused on paediatric populations. A pooled average estimate revealed a CSOM global prevalence of 3.8% of the global population, or 297 million people, 85% (252 million) of which in LMICs. 64 million (21.5%) of those affected had bilateral disease, and 184 million (62%) had disabling hearing loss defined as > 25–30 dB. Hearing impairment was reported in only four studies, which affected 50–78% of participants. Frequency of ear discharge was reported in only one study.

**Conclusion:**

The study identifies a significant global burden of CSOM, disproportionately affecting LMICs, and signals to healthcare providers and policymakers a pressing need for initiatives to prevent and manage this disease.

**Supplementary Information:**

The online version contains supplementary material available at 10.1007/s44197-025-00396-9.

## Key Points

**Question:**

What is the global prevalence of Chronic Suppurative Otitis Media (CSOM) and which variables are associated with variations in prevalence and sequelae?

**Findings:**

In this systematic review of 29 cross-sectional, community-based studies conducted between 2004 and 2024, the global prevalence of CSOM was estimated at 3.8%, affecting approximately 297 million people, with 85% of cases found in low- and middle-income countries. Bilateral disease was present in 21.5%, and 62% were associated with disabling hearing loss, defined as > 25–30 dB.

**Meaning:**

CSOM remains a significant global health issue, disproportionately affecting low- and middle-income countries, highlighting the urgent need for preventive and therapeutic interventions.

**Supplementary Information:**

The online version contains supplementary material available at 10.1007/s44197-025-00396-9.

## Introduction

Otitis media (OM) refers to inflammation of the middle ear and includes acute inflammation (acute otitis media, a middle ear infection) and chronic inflammation leading to middle ear effusion (chronic otitis media with effusion), retraction of the ear drum (tympanic membrane) or perforation of the ear drum (leading to chronic suppurative otitis media (CSOM). The exact pathophysiology of CSOM is unclear [1], but perforation of the tympanic membrane risks ear discharge (otorrhoea) and hearing loss. CSOM affects both children and adults, and contributes to stigma and reduced employment, education, wellbeing and quality of life with associated economic costs [2]. In sub-Saharan Africa and South-East Asia, targeted strategies for chronic otitis media rank among the most cost-effective interventions for reducing disability-adjusted life years (DALYs) [3].

Previous epidemiological reviews of CSOM are based on limited number of published literature. A 2004 study by the World Health Organization (WHO) estimated prevalence at anywhere between 65 and 330 million individuals [4], and a 2012 systematic review based on data up until 2008 estimated this at 200 million [5]. These studies may no longer reflect current disease burden, with the 2004 study based on data now more than 40 years old, and the 2012 study utilising a broad and imprecise definition of CSOM. Updated estimates can help guide healthcare resource allocation and policy interventions, particularly in low- and middle-income countries (LMICs) where CSOM is a major cause of preventable hearing loss.

Here we set out to provide an updated and methodologically robust estimate of the global prevalence of CSOM, underpinned by a systematic review of community based epidemiological studies from the last two decades. We sought to identify variables that are statistically associated with variation in disease prevalence and use these parameters (and only these parameters) to predict global epidemiology. We also sought to examine for evidence of sequelae of disease and use this to estimate global burden of consequences of disease.

## Materials and methods

We undertook a literature review in accordance with (PRISMA) guidelines [6]. The review was registered on PROSPERO with id CRD42024589635.

### Framework

- SPIDER (Sample, Phenomenon of Interest, Design, Evaluation, Research type) tool was used to structure the literature review, as it aligns with systematic reviews in qualitative and mixed-methods research [7, 8].
  1. **Sample**: Community-based CSOM populations.
  2. **Phenomenon of Interest**: Not applicable.
  3. **Design**: Potential modifiers of epidemiology.
  4. **Evaluation**: Global prevalence, incidence, and sequelae of CSOM.
  5. **Research Type**: Not applicable.

### Databases

- Embase and Medline.

### Search Strategy

- Search conducted on March 19, 2025.
- Search terms:
  - “chronic otitis media”, “chronic suppurative otitis media”, “middle ear inflammation”, “middle ear infection”, “purulent otitis media”, “tympanic perforation”.
  - Combined with: “incidence”, “prevalence”, “survey”, “epidemiology”, “proportion”, “risk factor”, “sequela”, “etiology”, “fatality”, “mortality”.
- Date restrictions: Limited to studies published between June 11, 2004 and March 19, 2025.
- Additional relevant studies were identified through snowballing reference lists of identified articles (Appendix I & II).

### Keywords Selection

- Keywords were selected to cover a comprehensive range of terms associated with CSOM, from basic terminology to specific clinical and epidemiological descriptors, ensuring maximal retrieval of relevant studies. Given variability in database indexing, we incorporated both free-text and medical subject heading terms to ensure comprehensive coverage.
- **“Chronic otitis media”**,** “chronic suppurative otitis media”**: Core terminology for identifying studies focused specifically on chronic inflammatory conditions of the middle ear.
- **“Middle ear inflammation”**,** “middle ear infection”**: Broader terms to capture studies that may use alternative terminology for CSOM.
- **“Purulent otitis media”**,** “tympanic perforation”**: Variants in clinical presentation to increase retrieval.
- **“Incidence”**,** “prevalence”**,** “survey”**,** “epidemiology”**,** “proportion”**: Epidemiological descriptors to focus on studies that report frequency and distribution of CSOM.
- **“Risk factor”**,** “sequela”**,** “etiology”**,** “fatality”**,** “mortality”**: Terms to capture data on CSOM risk factors, complications, and outcomes.

### Inclusion and Exclusion Criteria

- Inclusion Criteria:
  - Studies on human populations in community settings.
  - Articles reporting prevalence or incidence of CSOM in community populations.
  - Studies published June 11, 2004– March 19, 2025.
- Exclusion Criteria:
  - Older studies (prior to 2004) excluded to avoid outdated epidemiological data.
  - Hospital-based studies excluded due to potential selection bias, as they likely represent more severe cases rather than community prevalence.

### Data Extraction

Selection of studies and data extraction were managed using Covidence software (Covidence, Melbourne). Authors AO, HW and MB were involved in study selection and data extraction, and two authors (AO and HW) identified and independently assessed each full-text article to verify inclusion and extract relevant data. For each study we recorded title, lead author, location, year of publication, and study design. From each study we extracted data on:

- Epidemiology: study location, prevalence or incidence of disease, diagnostic criteria for CSOM (if recorded), and laterality of disease.
- Potential modifiers of epidemiology (where reported): including differentiated by age (< 18 years vs. > 18), sex (male vs. female), population type (rural vs. urban, indigenous vs. non-indigenous), other risk factors associated with disease prevalence.
- Symptoms and sequelae of disease: presence and severity of hearing loss, tinnitus or dizziness, frequency and severity of otorrhoea, and risk of mortality.

We critically evaluated these data to identify variables that associate with incidence or prevalence of CSOM and incorporated relevant variables to estimate global prevalence of disease. We extrapolated data on symptoms and sequelae to estimate global epidemiology of consequences of disease.

### Assessment of Level of Evidence and Quality

The quality of included studies was evaluated using an adapted version of the Joanna Briggs Institute (JBI) Checklist for Prevalence Studies [9]. Each study was scored from 0 (low quality) to 12 (high quality) using the following system:

1. Sample Frame Appropriateness (1 indicates partial or unclear, 2 indicates fully representative);
2. Recruitment Strategy (1 indicates unclear or unrepresentative, 2 indicates systematic and representative);
3. Sample Size (1 indicates insufficient or unjustified, 2 indicates adequate and justified);
4. Data Analysis Methods (1 indicates basic or incomplete, 2 indicates robust and appropriate);
5. Validity and Reliability of Diagnostic Criteria (1 indicates unclear or unstandardized, 2 indicates validated and standardized);
6. Response Rate (1 indicates low or not addressed, 2 indicates high or accounted for).

## Results

### PRISMA Diagram

**Fig. 1:**
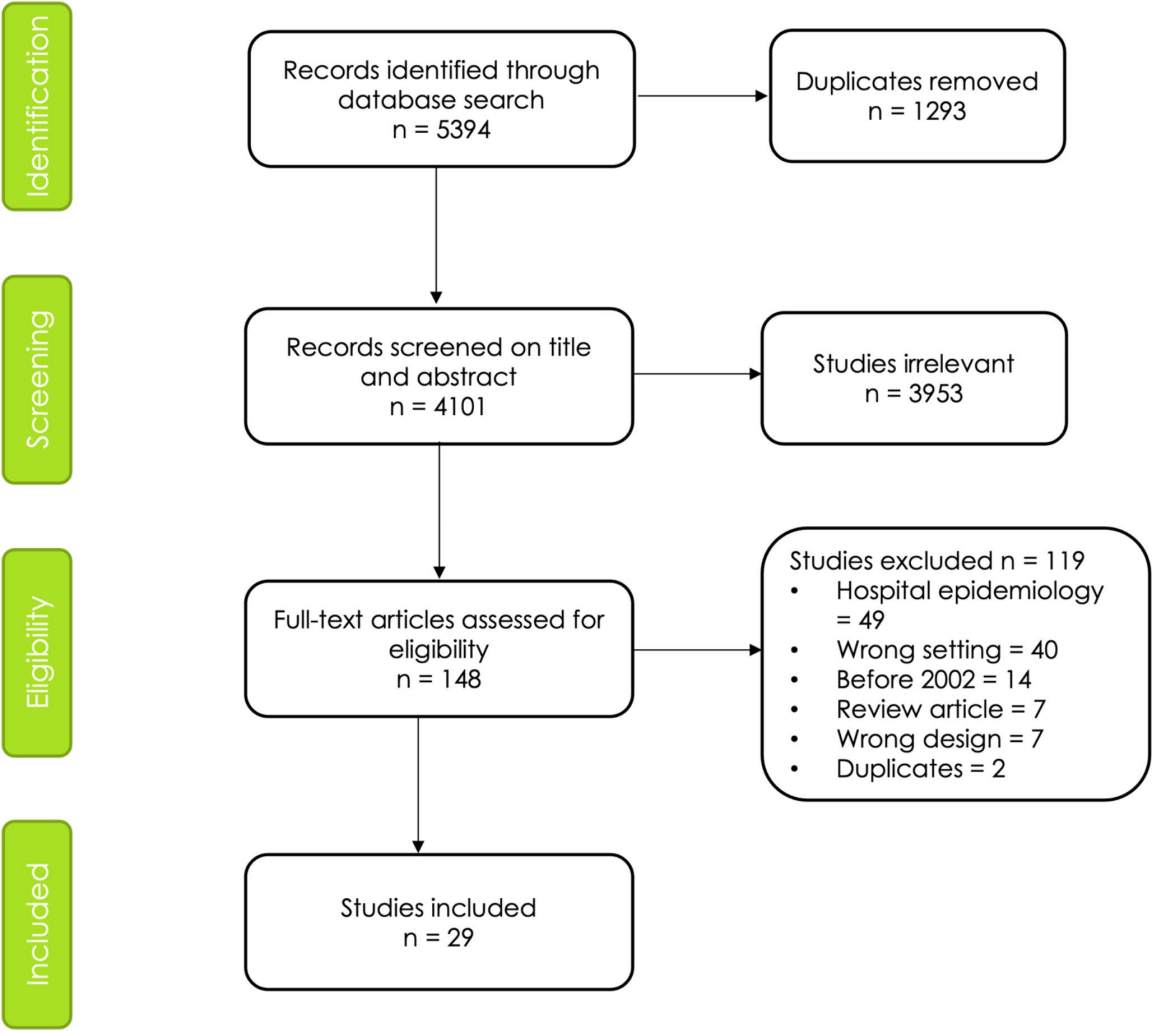
PRISMA diagram

The PRISMA diagram in Figure 1 illustrates the study selection process. The search found 5394 articles. After title/abstract and full-text screening, adopting selection criteria, and removing duplicates, 29 articles were eligible for inclusion. Any disagreements were resolved through discussion and consensus with the senior researcher. Table 1 lists included studies, listed by WHO region and income status of the country where they were undertaken.

**Table 1:** Prevalence of CSOM in community epidemiological studies. *Countries classified by WHO region*

Region/ Country	Income Status of country	Study	Score	Sampling frame	Study location	Ages included	Sample size	Prevalence % (*n*)
**Africa**								
Nigeria	Low	Amusa 2005 [29]	7	District	Ife Central Local Govt Area, Osun state	1 day– 12 yrs	600	2.5% (15)
Uganda	Low	Westerberg 2008 [32]	9	District	Masindi district	> 6 months	6,041	4.3% (260)
Kenya	Middle	Simoes 2016 [27]	11	Schools	9 districts of Kenya	2–15 yrs	13,109	1.5% (196)
Malawi	Low	Hunt 2017 [28]	7	District	Chikhwawa district	4–6 yrs	281	5.4% (15)
Rwanda	Low	Mukara 2017 [30]	9	District	Gasabo district, Kigali	6 months– 4.9 yrs	810	4% (32)
Cameroon	Middle	Libwea 2018 [25]	10	Community	6 districts of Yaoundé	2–3 yrs	433	0.7% (3)
Zimbabwe	Middle	Pedersen 2022 [34]	11	Schools	Mashonaland East province	4–13 yrs	451	0.4% (2)
**Eastern Mediterranean**								
Yemen	Middle	Muftah 2015 [33]	8	Schools	Socotra Island	6–16 yrs	686	7.4% (51)
**South-East Asia**								
Nepal	Low	Maharjan 2006 [16]	8	Schools	Morang district	5–15 yrs	1,050	6.2% (66)
	Low	Adhikari 2009 [18]	8	Schools	6 districts of Nepal	5–13 yrs	2,000	7.6% (153)
	Low	Adhikari 2009 [19]	9	Schools	4 districts of Nepal	5–15 yrs	500	5.0% (25)
	Middle	Maharjan 2020 [17]	9	Schools	Tibetan groups across Nepal	5–15 yrs	3,174	6.6% (209)
Bangladesh	Low	Shaheen 2012 [24]	8	Schools	Palash upazilla, Narsingdi district	4–12 yrs	1,468	5.2% (77)
	Middle	Shaheen 2014 [23]	8	Schools	Shibpur upazilla, Narsingdi district	5–12 yrs	1,645	3.7% (61)
India	Middle	Chadha 2013 [11]	9	Schools	New Delhi	5–12 yrs	15,718	4.8% (754)
	Middle	Chadha 2014 [12]	9	Districts	2 districts of Delhi	18 days– 15 yrs	3,000	4.3% (128)
	Middle	Chadha 2015 [13]	9	District	2 districts of Delhi	18 days– 15 yrs	4,626	3.6% (167)
	Middle	Parmar 2018 [14]	8	Schools	Muzaffarnagar, Uttar Pradesh	5–15 yrs	2,158	3.6% (49)
	Middle	Bellad 2019 [10]	7	Schools	Belagavi district (Hukkeri, Sankeshwar, Itagi), Karnataka	6–14 yrs	694	5.2% (29)
	Middle	Bright 2019 [15]	9	District	Northern Mahabubnagar District, Telangana	≥ 4 yrs	3,573	6.9% (246)
Indonesia	Middle	Anggraeni 2014 [26]	9	Schools	7 districts of Indonesia	6–15 yrs	7,005	1.7% (58)
**Western Pacific**								
South Korea	High	Cho 2010 [20]	11	Nationwide	Nationwide	2–80 yrs	4,930	2.8% (137)
	High	Park 2015 [22]	12	Nationwide	Nationwide	> 20 yrs	16,063	2.2% (349)
	High	Chung 2016 [21]	12	Nationwide	Nationwide	> 4 yrs	25,147	3.3% (832)
China	Middle	Bu 2011 [37]	11	Nationwide	Nationwide	All ages	29,246	0.9% (263)
Fiji	Middle	Fang 2016 [36]	6	District	Suva and Sigatoka areas (Korolevu, Cuvu, and Lomawai)	0–18 yrs	467	4.1% (19)
Solomon Islands	Middle	Kaspar 2018 [31]	7	Schools	Honiara	4–15 yrs	604	3.1% (18)
**Americas**								
Chile	High	Tamblay 2023 [35]	10	City	Santiago	≥ 50 yrs	538	3.5% (19)
**Europe**								
Greenland	High	Avnstorp 2016 [38]	7	Towns	Nuuk, Ilulissat and Maniitsoq	4–10 yrs	207	5.8% (12)

Income status determined by World Bank Status (37) at time of study (status only available up to 2021, so assumed to be the same as 2021 for later years)

CSOM = Chronic suppurative otitis media, n = number of cases, WHO = World Health Organisation, Yrs = years

### Study Details

#### Setting

**Fig. 2:**
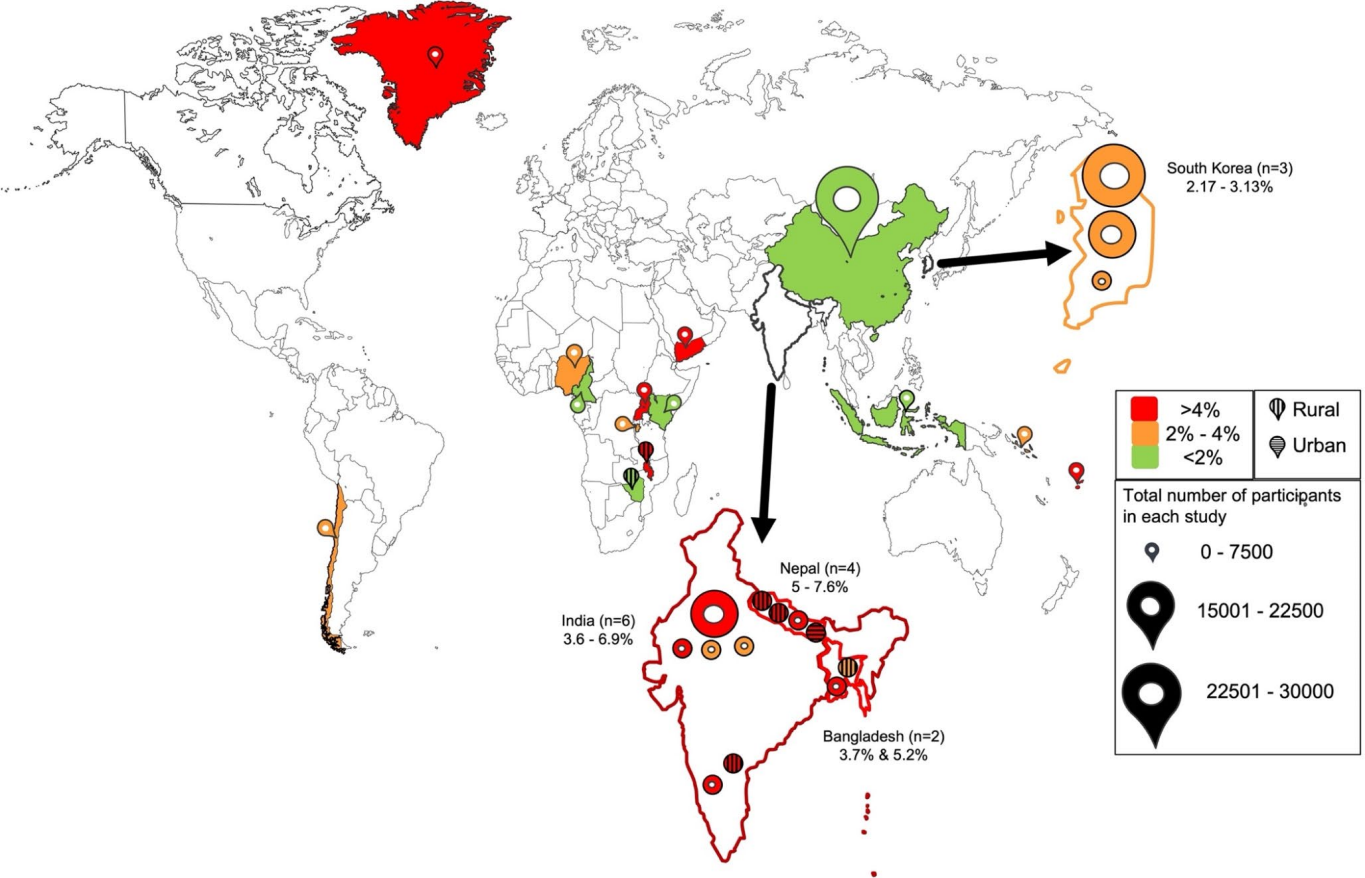
Map of CSOM prevalence. Prevalence category bounds follow those proposed by a 1996 WHO workshop [40]

Included studies were conducted between 2005 and 2023 in 18 countries, and encompassed surveys in schools as well as door-to-door visits. All included studies measured prevalence of CSOM; however, none assessed incidence. Studies took place in India (*n* = 6) [10–15], Nepal (*n* = 4) [16–19], South Korea (*n* = 3) [20–22], Bangladesh (*n* = 2) [23, 24], Cameroon [25], Indonesia [26], Kenya [27], Malawi [28], Nigeria [29], Rwanda [30], Solomon Islands [31], Uganda [32], Yemen [33], Zimbabwe [34]. Chile [35], Fiji [36], China [37], and Greenland [38]. Six studies [10, 16, 18, 24, 28, 34] were conducted exclusively in rural populations and one study [19] exclusively in an urban group (Figure 2).

**Sample size**: Sample sizes varied: 12 studies [10, 19, 25, 28–31, 33–36, 38] had fewer than 1,000 participants, 11 [12–18, 20, 23, 24, 27] between 1,000 and 5,000 participants, 2 [26, 32] between 5,000 and 10,000 participants, 2 [11, 22] between 10,000 and 20,000 participants, and 2 [21, 37] between 20,000 and 30,000 participants.

#### Outcome Details

Table 2 summarises definitions of CSOM used where applicable. Twelve studies [15, 16, 20, 22–24, 29, 31, 32, 35–37] did not specify the definition of CSOM used for diagnosis. Nine studies [12, 13, 25–27, 30, 33, 34, 38] defined CSOM according to the WHO criterion (persistent otorrhoea through a tympanic perforation for more than 2 weeks), one study [19] as persistent perforation of the tympanic membrane with or without otorrhoea of more than 3 months duration, and seven studies [10, 11, 14, 17, 18, 21, 28] as a permanent defect in the tympanic membrane without recording presence or duration of otorrhoea.

**Table 2:** Definitions of CSOM used in included studies

Definition of CSOM	Number of Studies
WHO criterion (persistent otorrhoea through a tympanic perforation for > 2 weeks)	9 [12, 13, 25–27, 30, 33, 34, 38]
Persistent perforation of the tympanic membrane with or without otorrhoea for > 3 months	1 [19]
Permanent defect in the tympanic membrane without recording presence or duration of otorrhoea	7 [10, 11, 14, 17, 18, 21, 28]
Not specified	12 [15, 16, 20, 22–24, 29, 31, 32, 35–37]

CSOM = Chronic suppurative otitis media

#### Epidemiology

Table 3 summarises subtypes of CSOM reported. Ten studies [12–14, 16, 19, 21, 24–26, 31] reported laterality of disease, with pooled analysis showing 21.5% of disease was bilateral (333/1549, standard deviation 2.9%). Eleven studies [10, 11, 14, 16, 18, 19, 21, 23, 24, 26, 27] separated disease into tubotympanic or atticoantral types, although we recognise this distinction of disease is contentious. Four studies (19,20,28,33) reported prevalence of cholesteatoma. Five studies [13, 16, 17, 21, 26, 27, 35] reported healed perforations and/or tympanosclerosis (prevalence between 0.1% and 4.2%), which study authors presumed to represent healed CSOM.

**Table 3:** Epidemiology of subtypes and laterality of CSOM. *Blank cells represent data not reported*

Study	Number of cases of CSOM (population prevalence)	CSOM subtype (population prevalence)			Proportion of cases bilateral % (*n*)
		Tubotympanic	Attico-antral	Cholesteatoma	
Maharjan 2006 [16]	66 (6.2%)	52 (5.0%)	14 (1.3%)	-	33% (22)
Adhikari 2009 [18]	153 (7.6%)	127 (6.3%)	26 (1.3%)	-	-
Adhikari 2009 [19]	25 (5%)	19 (3.8%)	6 (1.2%)	-	28% (7)
Shaheen 2012 [24]	77 (5.2%)	75 (5.1%)	2 (0.1%)	-	7% (5)
Chadha 2013 [11]	754 (4.8%)	617 (3.9%)	137 (0.9%)	-	-
Chadha 2014 [12]	128 (4.3%)	-	-	-	27% (34)
Shaheen 2014 [23]	61 (3.7%)	59 (3.6%)	2 (0.1%)	-	-
Anggraeni 2014 [26]	116 (1.66%)	114 (1.62%)	2 (0.03%)	0 (0%)	25% (29)
Chadha 2015 [13]	167 (3.6%)	-	-	-	25% (42)
Chung 2016 [21]	832 (3.3%)	448 (1.8%)	384 (1.5%)	83 (0.34%)	19% (172)
Simoes 2016 [27]	196 (1.5%)	185 (1.4%)	11 (0.1%)	6 (0.04%)	-
Libwea 2018 [25]	42 (0.7%)	-	-	-	7% (3)
Kaspar 2018 [31]	18 (3.1%)	-	-	-	16% (3)
Parmar 2018 [14]	78 (3.6%)	68 (3.2%)	10 (0.4%)		21% (16)
Bellad 2019 [10]	36 (5.2%)	29 (4.2%)	7 (1.0%)		
Tamblay 2023 [35]	19 (3.5%)	-	-	1 (0.1%)	
Mean average		74.9% (1792/2394)	25.1% (601/2394)	7.7% (90/1163)	21.5% (333/1549)

CSOM = Chronic suppurative otitis media, n = number of cases

### Evidence Associating Prevalence and Income Status

#### Outcome of Interest

We explored whether it was appropriate to estimate global prevalence using WHO region, income status, age, sex, population type, or putative risk factors as variables, by calculating pooled estimates from included studies along these categories.

**Analysis by WHO Region**: For three of the six WHO regions we only had data from one study (Table 1). For the other three, mean average prevalence in Africa was 2.4% (523/21,725, 95% Confidence Interval (CI): 2.2–2.6%, 7 studies), in South-East Asia 4.3% (2,022/46,611, 95% CI: 4.1–4.5%, 13 studies), and in Western Pacific 2.1% (1,618/76,457, 95% CI: 2.0–2.2%, 6 studies). Overall, there were insufficient studies from all WHO regions to provide confidence that calculating a pooled estimate on WHO regions was valid, although the data suggest there may be greater prevalence of CSOM in the South-East Asia region compared to Africa and Western Pacific.

**Analysis by income group**: When grouped by income level [39] (Table 4), mean average prevalence for low-income countries was 5.0% (641/12,750, 95% CI: 3.8– 6.2%, 8 studies), for middle income countries was 3.7% (3,158/86,589, 95% CI: 2.6– 4.8%, 16 studies), and for high income countries was 3.5% (1,646/46,885, 95% CI: 2.5– 4.6%, 5 studies). The prevalence in different income groups was highly significant (*p* < 0.0001, one-way ANOVA).

**Table 4:** Prevalence of global CSOM modelled to world bank income group

Income group	Mean prevalence rate (95% CI)	World bank population 2022 [47]	Individuals affected (95% CI) Rounded to nearest 100,000	Proportion of total disease (SD)
High	3.5% (2.5– 4.6%)	1,244,364,814	43,700,000 (17,200,000–70,200,000)	14.7% (+/- 5.8%)
Middle	3.7% (2.6 − 4.8%)	5,974,552,340	218,100,000 (132,100,000–304,100,000)	73.4% (+/- 44.5%)
Low	5.0% (3.8 − 6.2%)	703,727,949	35,400,000 (10,700,000–60,100,000)	11.9% (+/- 3.6%)
Total	3.8% (3.1 − 4.5%)	7,922,645,103	**297**,**200**,**000 (248**,**600**,**000 -345**,**800**,**000)**	

CSOM = Chronic suppurative otitis media, CI = Confidence interval, SD = Standard deviation

**Analysis by other variables**: Table 5 summarises distribution of CSOM cases by age, gender, and rural vs. urban populations. Five studies examined prevalence across different age groups, with no consistent finding: two [21, 33] studies reported a general increase in prevalence with age, and three [10, 18, 24] reported a general decrease in prevalence. Seven studies [10, 18, 21, 23, 24, 28, 33] examined sex differences, of which four [10, 18, 28, 33] found no difference and three [21, 23, 24] found female predominance, but with two of these studies [23, 24] having small sample size. Six studies [12, 14, 21, 23, 26, 33] measured CSOM in rural versus urban populations, where four [14, 21, 23, 26] reported higher prevalence in rural compared to urban, one [33] found higher prevalence in urban, and one [12] found varying results (a higher prevalence in rural compared to urban non-slum areas but a lower prevalence in rural compared to urban slum areas). There were no studies comparing indigenous to non-indigenous populations. Table 6 summarises possible risk factors for CSOM explored in seven included studies [10, 14, 21, 23, 28, 30, 33], but no studies compared presence of these factors to a control population, rendering findings unproven. One study [28] examined association between pneumococcal vaccination and CSOM and found no significant association. In summary, there was no evidence of a consistent association between disease prevalence and age, sex, population type, or putative risk factors. Such variables were therefore excluded from subsequent analysis.

**Table 5:** Reported prevalence of CSOM by age, gender and rural vs. urban populations (where reported)

Study	Number of cases of CSOM	Age *n* (%)	sex *n* (%)	Rural vs. Urban *n* (%)
Adhikari 2009 [18]	153	5-7yrs 76 (3.8%) 8-10yrs 43 (2.2%) 11-13yrs 34 (1.7%)	82 M (7.5%) 71 F (7.8%)	--
Shaheen 2012 [24]	77	< 6yrs 2 (15.4%) 6-8yrs 28 (4.6%) > 9yrs 47 (5.6%)	46 F (5.7%) 31 M (4.7%)	-
Shaheen 2014 [23]	61	-	Rural 18 F (6.0%) 23 M (6.0%) Urban 11 F (2.3%) 9 M (1.8%)	Rural 41 (6.0%) Urban 20 (2.1%)
Anggraeni 2014 [26]	58	-	-	Rural 91 (2.7%) Urban 25 (0.7%)
Chadha 2014 [12]	128	-	-	Rural 30 (3%) Urban Slum 72 (7.2%) Urban Non-slum 26 (2.6%)
Muftah 2015 [33]	51	< 10yrs 21 (6.7%) > 11yrs 30 (8.1%)	23 F (7.1%) 28 M (7.0%)	Rural 23 (6.8%) Urban 28 (8.1%)
Chung 2016 [21]	832	4-12yrs 0.55 (10.1%) 12-18yrs 0.37 (9.1%) 19-29yrs 1.01 (9.8%) 30-39yrs 1.75 (15.3%) 40-49yrs 3.06 (15.1%) 50-59yrs 5.60 (15.2%) 60-69yrs 6.69 (13.4%) ≥ 70yrs 8.52 (12.1%)	488 F (3.5%) 365 M (2.8%)	Rural 2,827 (4.1%) Urban 11,569 (3.2%)
Hunt 2017 [28]	15	-	6 F (5.1%) 9 M (5.4%)	-
Parmar 2018 [14]	49	-	-	Rural 51 (5.1%) Urban 27 (2.3%)
Bellad 2019 [10]	29	≤ 10yrs 5 (9.8%) ≥ 11yrs 31 (4.9%)	17 F (5.3%) 19 M (5.1%)	-

CSOM = Chronic suppurative otitis media, F = female, M = male, n = number of cases, % = percentage count within group

**Table 6:** Risk factors explored for association with CSOM in included studies (none proven to be statistically significant)

Study ID	Study size	Risk factors explored
Shaheen 2014* [23]	61	• Parental occupation and annual income • Housing type • Family size > 6 members • Maternal education • Bathing habit
Muftah 2015* [33]	51	• Parental illiteracy • Overcrowding • Swimming in local water pools • URTI
Chung 2016*^^Σ^ [21]	25,147	• Alcohol consumption • Diabetes • Single house living • Maternal education
Hunt 2017 [28]	15	• Sleeping in household > 2 other children • Vaccination status
Mukara 2017*^ ^Σ^ [30]	810	• Household smoke • Parental education
Parmar 2018 [14]	78	• Social class • Paternal smoking • Indoor / Kerosene oil cooking • URTI, chronic tonsillitis, adeno-tonsillitis
Bellad 2019 [10]	69	• Recurrent respiratory infection • Ear cleaning • Overcrowding • Maternal Illiteracy

CSOM = Chronic suppurative otitis media, ID = identification, URTI = upper respiratory tract infection

### Epidemiology of Sequelae of CSOM

Epidemiology of otorrhoea was reported in only one study of children in Kenya [27], which found that in those with ear discharge (*N* = 901), onset was by the age of 3.5 years in half of cases, and by age of 6 years in three-quarters. No study reported frequency or severity of otorrhoea.

Hearing impairment was recorded in seven studies [15, 17, 28, 31, 33, 34, 38], but three of these [15, 34, 38] only included average data, which meant they were not informative for calculating the population proportion with hearing loss. Data from the remaining four studies [17, 28, 31, 33] are summarised in Table 7. Where thresholds were reported these were either three [33] or four [17] tone averages on pure tone audiometry. Pooling data from these four studies and defining hearing loss as thresholds > 25-30dB (where measured), 62% (182/293, SD 0.49) of those with CSOM have hearing loss. This is consistent with data from prior reviews [41].

A multivariate analysis conducted in one study [21] found a significant association between presence of tinnitus and CSOM in participants aged over 19 years but did not report prevalence of this symptom. No studies reported prevalence of dizziness in those with CSOM. No studies reported mortality rates.

**Table 7:** Association of CSOM with hearing impairment

Study ID	Number of cases of CSOM	Proportion of CSOM cases with hearing impairment	Level of hearing loss *n* (%)
Muftah 2015 [33]	51	67% (34)	< 30 dB 17 (33%) Mild loss (31–40 dB) 19 (37%) Moderate loss (41–60 dB) 13 (26%) Severe loss (61–80 dB)2 (4%) Profound loss (> 80 dB) 0 (0%)
Hunt 2017 [28]	15	67% (10)	Not specified
Kaspar 2018 [31]	18	78% (14)	Not specified
Maharjan 2020 [17]	209	59% (124)	< 25 dB: 45 (5%) Mild CHL (26–40 dB): 135 (75%) Moderate CHL (41–60 dB): 25 (17%) Severe CHL (61–80 dB): 1 (0.7%) Moderate mixed loss (41-60dB): 2 (1.5%) Severe mixed loss (61-80dB): 1 (0.7%)

CHL = conductive hearing loss, CSOM = Chronic suppurative otitis media, dB = decibels hearing level, n = number of cases

### Estimated Global Epidemiology of CSOM

Based on our data review we calculated pooled estimates for the global prevalence of CSOM focusing exclusively on country income levels as the only variable found to correlate with disease prevalence. We mapped prevalence to the global population living in each of those country income levels (Table 4). Overall, we estimate over 297 million (95% CI: 248 million– 345 million) people in the world have CSOM. On the assumption of an event rate of 21.5% (Table 3) this implies 64 million of these have bilateral disease, and on the assumption of an event rate of 62% (Table 7) 184 million have hearing loss.

## Discussion

Our review has established a global CSOM prevalence of 3.8%. Of those affected, 85% live in LMICs, with the highest prevalence found in low-income countries. This principal finding confirms CSOM as a major global disease, and emphasizes the need for heightened awareness, and a need for increased health resources and public health approaches to combat disease. Beyond its clinical impact, CSOM also carries economic implications (particularly in LMICs), through healthcare treatment costs, chronic disability affecting employment and education.

Our findings are consistent with previous estimates of prevalence, where Acuin estimated global prevalence of CSOM at between 65.5 and 328.2 million [4], using data published data between 1966 and 1997. Those estimates were modelled by extrapolation of data within a WHO region to the whole region, but in this study, there was an up to a 13-fold difference in prevalence within a region, explaining the wide range in the estimated global prevalence and suggesting that a geographical basis for modelling may not be justified. In addition, this analysis was not strict in its definition of disease and the paper recognised likely diagnostic misattribution in some of the studies included. A study by Monasta similarly extrapolated sparse regional data, but also did not define a review protocol, did not list the studies included, and did not include a subject expert in study selection, making it difficult to verify that the selection process was appropriate [5]. The strength of our review is our use of recent data (within the last 20 years) and estimating prevalence using country income group as the only parameter we found to be statistically associated with prevalence. Our approach has also enabled greater precision to estimates of prevalence when compared to previous studies.

We found few studies on the consequences of CSOM, and future studies on community epidemiology should look to measure the presence and severity of such sequelae and include access to raw data (for example on audiological thresholds) to enable analysis by other researchers. For hearing loss there were only four informative studies in our review, and there were no studies reporting data on otorrhoea, dizziness or tinnitus. Studies of hospital populations (which may represent disease more severe than that found in community) report that in 391 children with CSOM attending a hospital in Addis Ababa (Ethiopia), otorrhoea was continuous in 27% and recurrent in 73% [42], and in 231 adults with CSOM attending specialist hospitals in Colombia, 52% reported severe tinnitus, 22% moderate tinnitus, and 27% minor [43], and that up to 65% of the same population reported some symptoms of dizziness [44].

Our review has limitations. It was based upon studies from only 19 countries or regions, and of these two focused on particular populations: one study from Nepal recruited only Tibetan groups [17], and the study from Greenland only Inuit populations [38] (who like many indigenous groups, are known to have high prevalence of CSOM) [45]. There was heterogeneity between studies, including differences in age groups, sampling methods, and sample sizes. These data may therefore not be representative of other geographical locations. Our analysis did not find evidence of variation in prevalence by age, gender, or urban versus rural settings, but given that only a few studies examined this and were methodologically flawed in that they did not compare to a control group, we cannot exclude that such variations do in fact exist. Only one study [28] assessed association of vaccination status with CSOM, but this could be an important variable affecting disease prevalence and so is worthy of further studies.

Our estimate of the prevalence of disabling hearing loss associated with CSOM is based upon only four studies, and may be an over-estimate, because here we defined loss as pure tone average thresholds greater than 25-30dB to match to data within studies, whereas evidence shows speech performance noticeably deteriorated at thresholds > 35dB [46], which is classified as moderate loss on the WHO grading system [47]. There was variability between studies in how CSOM was defined, which complicates comparisons across studies. This challenge is recognized by the academic community to affect the validity of reported prevalence estimates, and our group is currently working to develop global consensus on definition of CSOM for future epidemiological studies and clinical trials.

In conclusion, we estimate that around 1 in 26 of the global population, or over 297 million people suffer from CSOM: 64 million with bilateral disease, and 184 million with associated hearing loss. There is a need to explore and evidence approaches to tackle this large disease burden, for example through community-based approaches to treating ear discharge, exploiting low-cost technology for hearing rehabilitation, and expanding capacity for surgical repair of the eardrum [1].

## Electronic Supplementary Material

Below is the link to the electronic supplementary material.

Supplementary Material 1

## Data Availability

No datasets were generated or analysed during the current study.

## Declarations

### Use of Artificial Intelligence (AI) Tools

No AI tools were used.
