# Supplementary figures and images for: Epidemiology of Chronic Suppurative Otitis Media: Systematic Review To Estimate Global Prevalence
Source: J Epidemiol Glob Health. 2025 Apr 3;15(1):55. doi: 10.1007/s44197-025-00396-9 (PMC11968643; doi:10.1007/s44197-025-00396-9)

# Supplementary material Appendix I: Initial Embase search

*
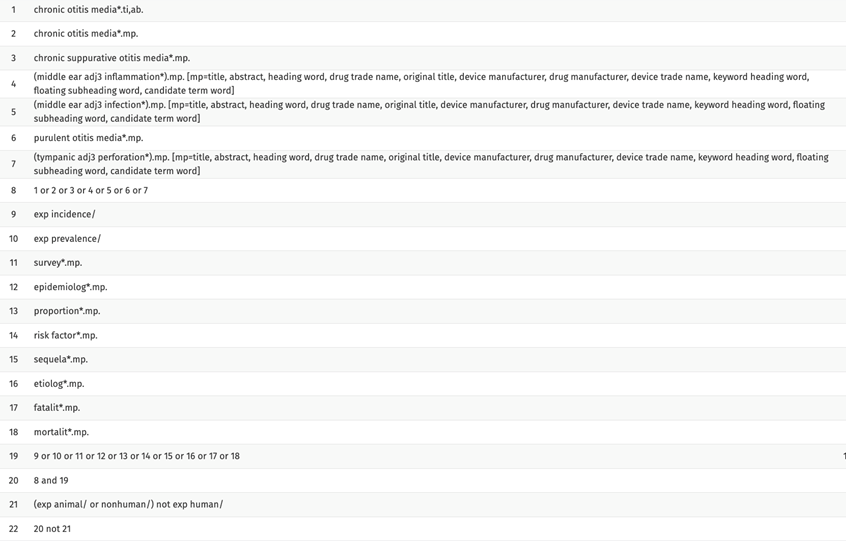
*

## Appendix II. Initial Medline search

*
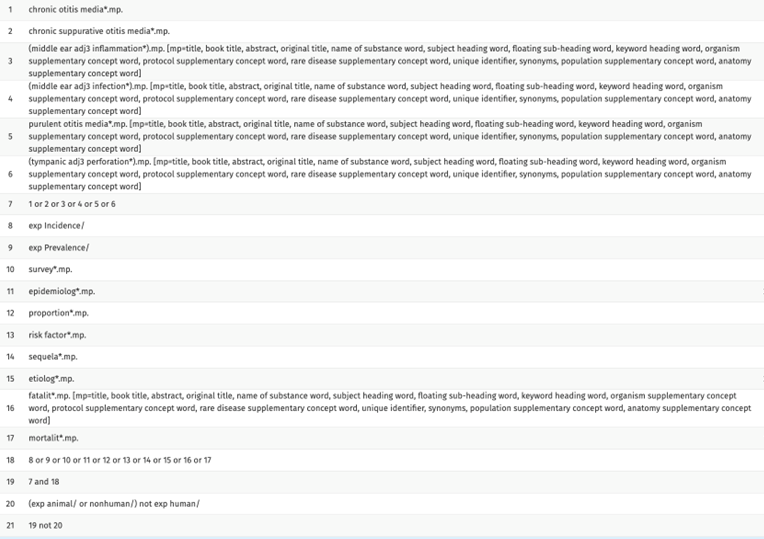
*

Supplement: Supplementary file 1 — Supplementary Material 1 [file 44197_2025_396_MOESM1_ESM.docx]
